# Efficacy and Safety of Terlipressin Infusion in Hepatorenal Syndrome-Acute Kidney Injury (HRS-AKI): A Retrospective Observational Study

**DOI:** 10.7759/cureus.66581

**Published:** 2024-08-10

**Authors:** Manoj Gowda, Dave Manan Dilipbhai, Umesh Jalihal, Madduri Pavan Kumar, Bharath Gowda S, Anil Jain, Naveen Ganjoo

**Affiliations:** 1 Medical Gastroenterology and Hepatology, Sapthagiri Institute of Medical Sciences and Research Centre, Bengaluru, IND; 2 Hepatology, Aster RV Hospital, Bengaluru, IND

**Keywords:** hrs-aki, hepatorenal syndrome-acute kidney injury, hepatorenal syndrome, chronic liver disease (cld), acute kidney injury, albumin, terlipressin infusion

## Abstract

Background

Hepatorenal syndrome-acute kidney injury (HRS-AKI) is an event that occurs in chronic liver disease (CLD) and is associated with high morbidity and mortality. Terlipressin, a vasopressin analog, is used for the treatment of portal hypertension-related gastrointestinal (GI) bleeding and is found to be effective in the management of HRS-AKI. Continuous infusion of terlipressin maintains a high mean arterial pressure while reducing adverse events. It is better tolerated and equally effective at lower doses than intravenous boluses in patients with HRS-AKI.

Aim of the study

This study aimed to evaluate the safety and efficacy of terlipressin infusion at the rate of 4 mg/day in the treatment of HRS-AKI.

Methods

This retrospective study included patients who had HRS-AKI according to the modified International Club of Ascites (ICA) definition. Patients were started on a continuous intravenous infusion. The included patients received terlipressin 1 mg stat followed by a 4 mg infusion over 24 hours, and the infusion was continued until specific response criteria were met or for a maximum of seven days.

Results

In total, 136 patients were included in this study. The mean age of the study group was 45 years, the mean Child-Turcotte-Pugh (CTP) score was 11, the mean model for end-stage liver disease (MELD) score was 30, and the mean serum creatinine was 2.46 mg/dl. A response to treatment in the form of reduction of serum creatinine was observed in 94 (69.1%) patients, 30 (22%) patients showed no response, and worsening of creatinine was seen in 12 (8.8%) patients. The mean duration of hospital stay was 7.6 days, the mean serum creatinine was 1.17 mg/dl at the end of treatment, and the mean CTP and MELD scores in treatment responders were nine and 27, respectively. A total of 29 (21.3%) of 136 patients had adverse events during the terlipressin infusion therapy.

Conclusion

Terlipressin infusion has sustained effects on splanchnic hemodynamics with fewer and less severe adverse events than intravenous bolus doses. Terlipressin infusion at a dose of 4 mg/day appeared to be well tolerated, with similar outcomes to that of 2 mg/day with a significantly lower albumin dose. These findings emphasize the importance of optimizing treatment protocols, particularly those favoring infusion methods, to enhance efficacy and minimize adverse effects.

## Introduction

Hepatorenal syndrome-acute kidney injury (HRS-AKI) is a sequence of events occurring in the setting of chronic liver disease (CLD), which is associated with splanchnic vascular dilatation, leading to significant renal arterial vasoconstriction and progressive renal failure [1]. There are no histological changes in the kidneys in the initial phase, and prompt management of portal hypertension normalizes renal function [2,3]. The annual frequency of HRS-AKI in cirrhotic patients with ascites is 8%-40% [4]. Upon the establishment of HRS-AKI, both morbidity and mortality remain high, which has led to a focus on the prevention, early diagnosis, and therapy of renal dysfunction in patients with cirrhosis.

The diagnostic criteria for HRS-AKI proposed by the International Club of Ascites (ICA) were modified in 2019, and urine output was added to the existing criteria [5]. Treatment of HRS-AKI consists of discontinuation of nephrotoxic agents, antibiotics for infection, and volume expansion by intravenous albumin and vasopressor therapy. Terlipressin, a vasopressin analog used for the treatment of portal hypertension-related gastrointestinal (GI) bleeding, is found to be effective in the management of HRS-AKI. The distribution half-life of terlipressin is eight minutes, and the peak concentration occurs around 10 minutes after intravenous bolus administration. Endothelial peptidases cleave terlipressin, causing lysine vasopressin to be released gradually over four to six hours [6-8].

The recommended dose of terlipressin is 1 mg intravenously every four hours, to begin with, and increases up to 2 mg every four hours if the baseline serum creatinine level does not improve by 25% on day three of therapy. The effect of terlipressin on splanchnic hemodynamics, such as portal pressure, wears off three to four hours after intravenous administration in individuals with cirrhosis. Terlipressin reached its peak concentration 10 min after intravenous bolus administration. However, the current intravenous terlipressin bolus protocol suggests a four- to six-hour interval between bolus doses. Theoretically, the drug can improve arterial splanchnic hemodynamics but should not be able to maintain a stable state for 24 hours [9]. Continuous infusion of terlipressin has been found to maintain a high mean arterial pressure while reducing adverse events; however, it is important to note that it may still be associated with severe adverse effects in patients with a high model for end-stage liver disease (MELD) score [10]. This study aims to establish the efficacy, safety, and adverse effects of continuous terlipressin infusion in the treatment of HRS-AKI.

## Materials and methods

In this retrospective study, patients with HRS who received terlipressin as an infusion between September 2022 and March 2024 were identified. Using the hospital database, a total of 165 patients were identified who were admitted to the Sapthagiri Institute of Medical Sciences and Research Centre, Bangalore, India, between September 2022 and March 2024 with decompensated CLD, portal hypertension, and HRS and received terlipressin infusion to reduce portal pressure and improve renal perfusion.

After obtaining clearance from the Sapthagiri Institute of Medical Sciences and Research Centre's institutional ethics committee (approval number: 09/SS-08/2024-25), the data were analyzed using IBM SPSS Statistics for Windows, version 27.0 (IBM Corp., Armonk, NY) and the SAS statistical package, version 9.4 (SAS Inc., Cary, NC).

Out of 165 cases, 29 were found to be ineligible with the inclusion criteria and were excluded from the study. Of these 29 patients, two were aged >70 years, two were diagnosed with hepatocellular carcinoma, two had chronic kidney disease, seven had diabetes, two had severe hypertension, nine had sepsis, and five had hyponatremia <120 mEq/l.

Patients who met the HRS-AKI criteria, according to the modified ICA definition, were included. Diuretic medications were withheld, and an albumin infusion (20 g/day) was administered for plasma expansion. Patient demographics, clinical information, laboratory results, vital signs, and prognostic scores were collected from available data. Physical examination, electrocardiogram, chest radiography, and standard laboratory tests conducted during the therapy were compared. The included patients received terlipressin 1 mg stat followed by a 4 mg infusion over 24 hours and continued until specific response criteria were met or for a maximum of seven days.

Inclusion criteria

Inclusion criteria and exclusion criteria are defined in Table 1 and Table 2, respectively.

**Table 1:** Inclusion criteria GI: gastrointestinal; AKI: acute kidney injury; ICA: International Club of Ascites; HRS: hepatorenal syndrome

Inclusion criteria
Age >18 years
Cirrhosis demonstrated by clinical, biochemical, imaging, or liver biopsy, with additional information from an upper GI endoscopy
Diagnosis of AKI according to the ICA criteria; diagnosis of Type 1 HRS according to the ICA criteria

**Table 2:** Exclusion criteria

Exclusion criteria
Septic shock
Evidence of recent use of nephrotoxic agents, absence of intrinsic renal disease
Hepatocellular carcinoma
Cardiac, respiratory failure, or serious extrahepatic illness
Contraindications to terlipressin
Hyponatremia <120 mEq/l

Response to therapy

Response to therapy has been divided into complete and incomplete responses as shown in Table 3. 

**Table 3:** Response to therapy AKI: acute kidney injury

Response to therapy	
Complete response	Defined as a decrease in serum creatinine to <1.5 mg/dl from the baseline value at admission
Partial response	Defined as a fall in the AKI stage of at least one with serum creatinine >0.3 mg/dl above the baseline value [11]

Objectives

The objectives of the study are defined in Table 4.

**Table 4:** Objectives of the study

Objectives	
Primary endpoint	Assessment of response to therapy
Secondary endpoints	Safety of therapy measured by the frequency of drug-related adverse events

## Results

Using the hospital database, a total of 165 patients who were admitted to the Sapthagiri Institute of Medical Sciences and Research Centre between September 2022 and March 2024 with decompensated CLD, portal hypertension, and hepatorenal syndrome and received terlipressin infusion to reduce portal pressure and improve renal perfusion were identified.

**Figure 1:**
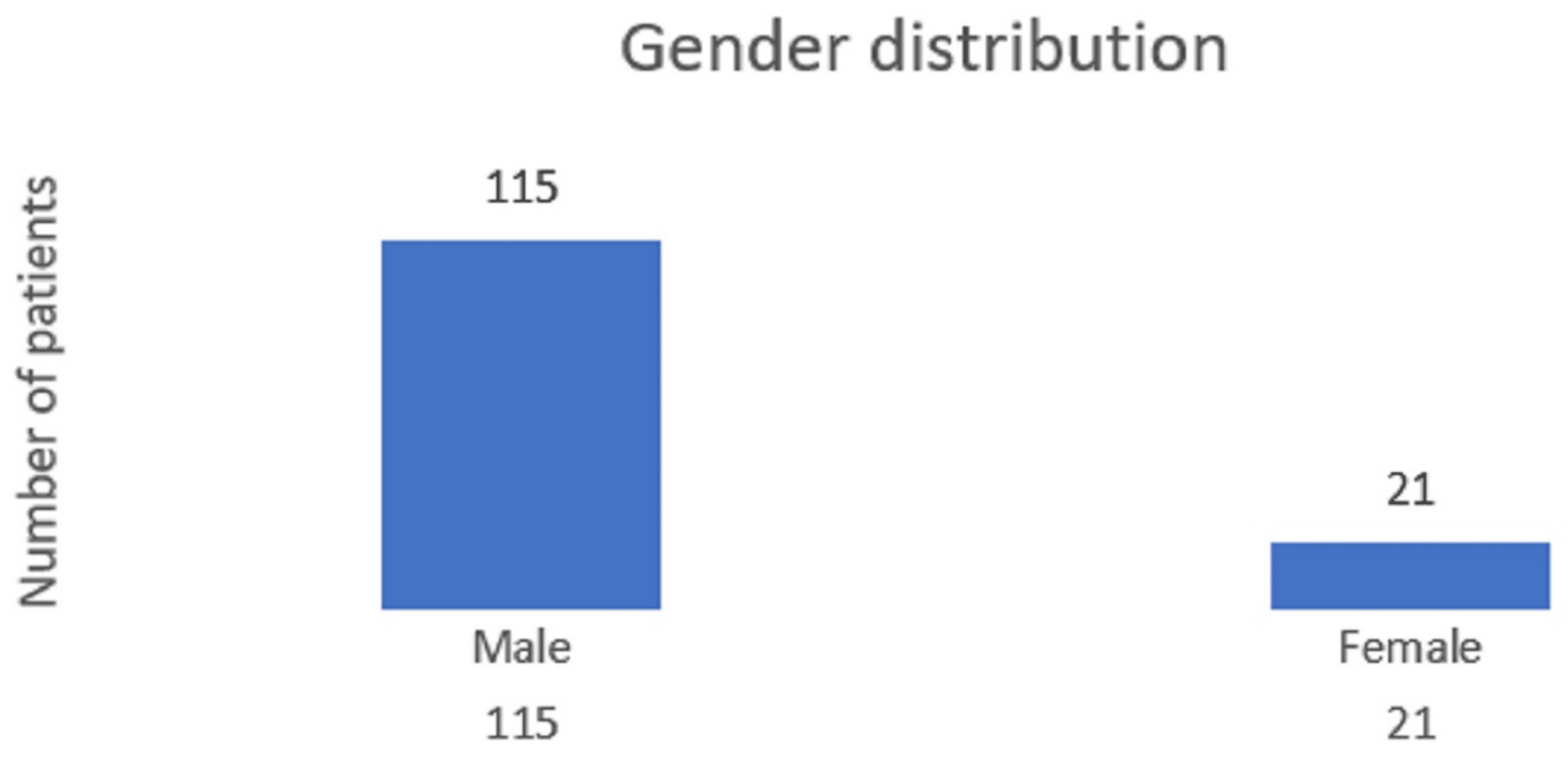
Distribution of patients according to gender

**Figure 2:**
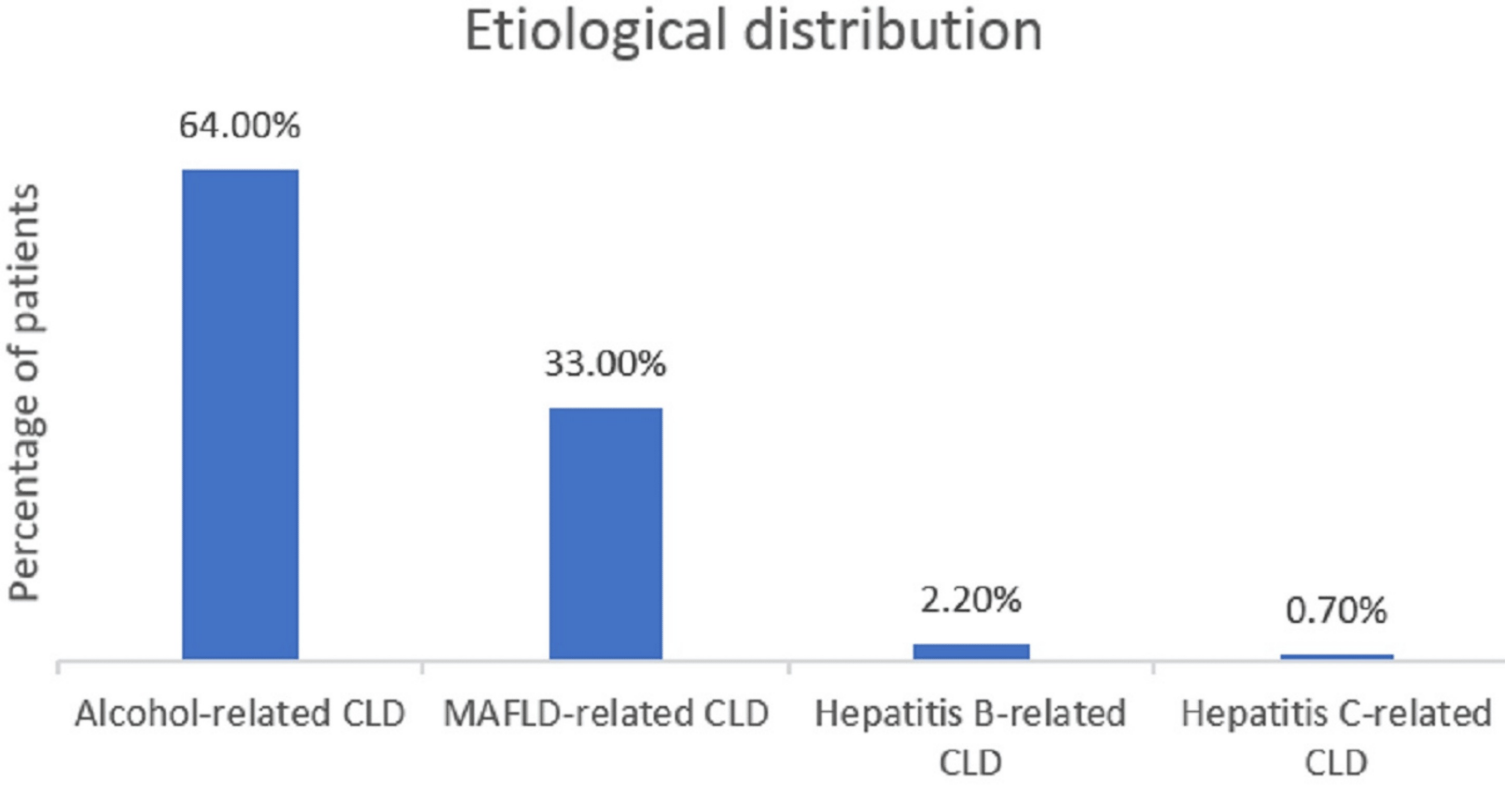
Distribution of patients according to their etiology CLD: chronic liver disease; MAFLD: metabolic dysfunction–associated fatty liver disease

A total of 136 patients who fulfilled the inclusion criteria were included in this study. The mean age of the patients in the study group was 45 years. Of the 136 patients, 115 (84.4%) were male and 21 (15.4%) were female, as shown in Figure 1. Eighty-seven (64%) patients had alcohol-related CLD, 45 (33%) had nonalcoholic steatohepatitis (NASH)-related CLD, three (2.2%) had hepatitis B-related CLD, and one (0.7%) had hepatitis C-related CLD, as shown in Figure 2. The mean Child-Turcotte-Pugh (CTP) score was found to be 11, the mean MELD score was found to be 30, and the mean serum creatinine at admission was 2.46 mg/dl in the study group. The mean values of the liver function test at admission are as follows: the mean total bilirubin was 6.8 mg/dl, the mean aspartate aminotransferase (AST) was 218 IU/l, the mean alanine transaminase (ALT) was 182 IU/l, the mean serum protein was 6.2 g/dl, the mean albumin was 2.77 g/dl, and the mean serum sodium was 130 mEq/l, as shown in Table 5.

**Table 5:** Baseline parameters of patients with hepatorenal syndrome–acute kidney injury

Blood investigation	Patient values
Hemoglobin (g/dl)	9.2
Total leucocyte count (× 10^3^/l)	8.8
Platelet count (× 10^9^/l)	92
Total bilirubin (mg/dl)	6.8
Direct bilirubin (mg/dl)	4.5
Aspartate aminotransferase (IU/l)	218
Alanine transaminase (IU/l)	182
Alkaline phosphatase (IU/l)	137
Gamma-glutamyl transferase (IU/l)	80
Albumin (g/dl)	2.77
Protein (g/dl)	6.2
International normalized ratio	2
Serum creatinine (mg/dl)	2.46
Blood urea nitrogen (mg/dl)	87
Child–Turcotte–Pugh score	11
Model for end-stage liver disease score	30
Serum sodium (mEq/l)	130
Serum potassium (mEq/l)	4.8

**Figure 3:**
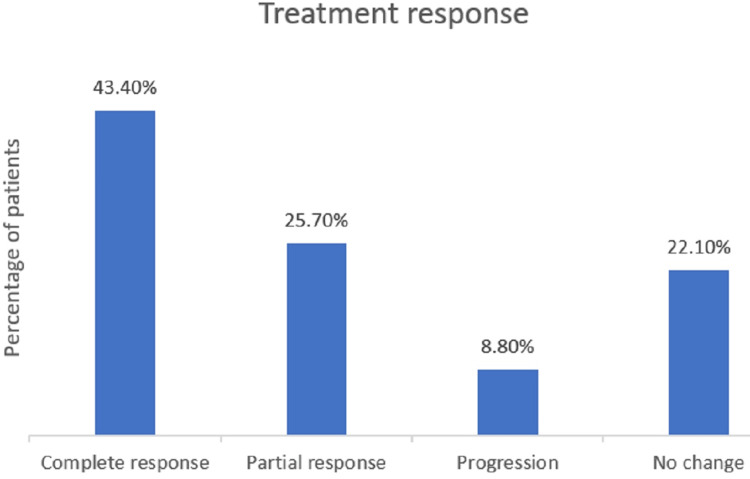
Treatment response of the patients

Response to treatment with terlipressin infusion in the form of reduction of serum creatinine was observed in 94 (69.1%) patients; serum creatinine in 30 (22%) patients remained the same as the admission level, whereas worsening of creatinine was seen in 12 (8.8%) patients. Details of the treatment response are shown in Figure 3. The mean duration of hospital stay was 7.6 days, the mean end-of-treatment serum creatinine was 1.17 mg/dl, the mean cumulative dose of albumin was 100 g, the maximum daily dose of terlipressin was 4 mg, and the mean CTP and MELD scores in treatment responders were nine and 27, respectively, as shown in Table 6. Of the 12 patients who had worsening creatinine levels, 11 had an alcohol-related CLD with a mean MELD score of 36, and one patient had a NASH-related CLD with a MELD score of 33.

**Table 6:** Details of the treatment responders

Details of treatment responders: mean values	n = 94
Duration of hospital stay (days)	7.6
End of treatment serum creatinine (mg/dl)	1.17
Cumulative dose of albumin (gm)	100
Maximum daily dose of terlipressin (mg)	4
Child–Turcotte–Pugh score	9
Model for end-stage liver disease score	27

**Figure 4:**
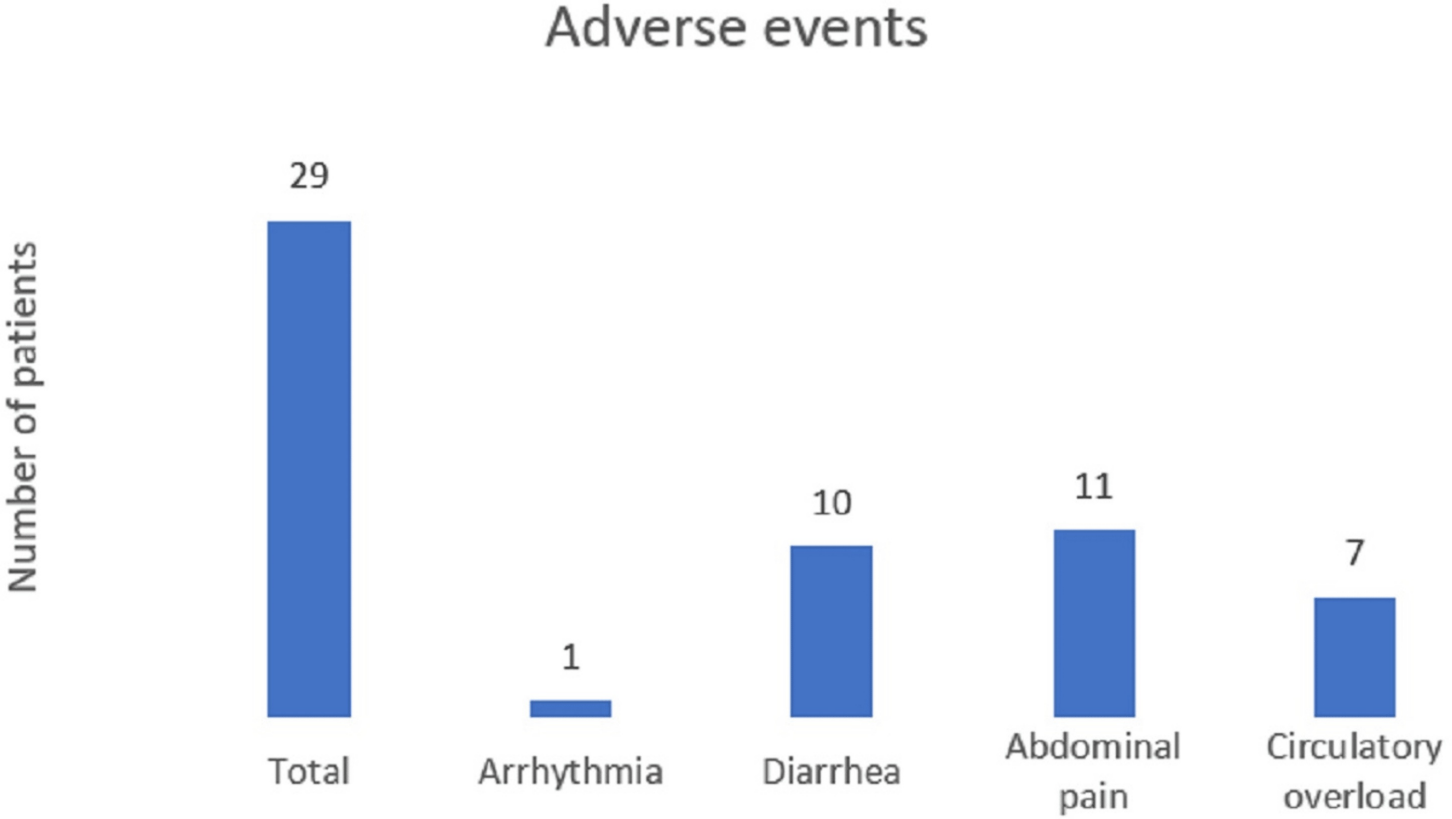
Adverse events noted among the patients

Twenty-nine (21.3%) of 136 patients had adverse events during terlipressin infusion therapy. Seven (5.1%) patients had circulatory overload, 10 (7.4%) had diarrhea, 11 (8%) had abdominal pain, and one (0.7%) had arrhythmia, as shown in Figure 4. Patients with diarrhea and abdominal pain were managed with a reduction in the infusion rate for a few hours. Once the symptoms subsided, they were again started at an infusion rate of 4 mg/24 hours. Initially, the infusion was stopped for patients developing circulatory overload, albumin was optimized, and the patients were restarted on infusion of terlipressin at 2 mg/24 hours, eventually reaching 4 mg/24 hours. Infusion was completely discontinued in the patients who developed arrhythmia. There was a mean reduction in serum sodium by 1.2 mEq/l; none of the patients developed severe hyponatremia requiring termination of terlipressin infusion.

## Discussion

This study provides insights into the demographic and etiological patterns of CLD in its cohort by comparing its findings with those of several other studies. In our study, the mean age of the participants was 45 years, with a significant preponderance of male patients (84.5% men and 15.4% women). The primary cause of CLD was alcohol-related, accounting for 64% of cases. Other etiologies included metabolic dysfunction-related fatty liver disease (33%), hepatitis B (2.2%), and hepatitis C (0.7%).

In our study, the mean CTP and MELD scores were 11 and 30, respectively. Studies with similar MELD and CTP scores, which included terlipressin administration for HRS-AKI, were analyzed and compared with our study. Cavallin et al. found that the mean CTP scores were 10.79 in the infusion group and 10.78 in the bolus group, with MELD scores of 29.2 and 29.8, respectively [12]. These scores are very similar to those of our study, suggesting a similar severity of liver dysfunction among the patients. In a study by Wong et al., the mean CTP score was 10 and the mean MELD score was 32.7 [13]. In a study by Gupta et al., the mean CTP score was slightly higher in the infusion group (12) than in the bolus group (10.45), while the mean MELD scores were 30.2 and 27.08, respectively [14]. Across all studies, the CTP and MELD scores reflected a high severity of liver disease.

In our study, the mean serum creatinine level was 2.46 mg/dl; in a study by Gupta et al. [14], the mean serum creatinine levels were 2.4 mg/dl in the infusion group and 2.1 mg/dl in the bolus group. These values are similar to our findings, indicating similar renal impairment. In addition, Cavallin et al. [12] reported that the mean serum creatinine levels were higher, at 3.4 mg/dl in the infusion group and 3.1 mg/dl in the bolus group, suggesting more severe renal dysfunction in their cohort compared to our study. Wong et al. reported the highest mean serum creatinine level of 3.5 mg/dl. Our study's mean creatinine level suggests significant but less severe renal dysfunction compared with the studies by Cavallin et al. [12] and Wong et al. [13], which reported levels >3 mg/dl.

In our study, the overall response rate was 69.1%, with 43.4% of patients achieving a complete response and 25.7% achieving a partial response. The mean CTP score among the responders was nine, and the mean MELD score was 27. In a study by Gupta et al. [14], the response rate was higher in the infusion group (76%) than in the bolus group (68.75%). The mean CTP score was 11.2, and the mean MELD score was 27.8 among responders in the infusion group. These scores indicate slightly more severe liver disease than in our study, yet with a comparable response rate. In the study by Cavallin et al. [12], the response rate in the infusion group was 76.5% (with 55.8% achieving complete response and 20.59% partial response). In the bolus group, the response rate was 64.9% (45.95% complete response and 18.9% partial response). These lower MELD scores compared with those in our study suggest that their patients may have had slightly less severe liver disease. In a study by Wong et al. [13], the response rate was notably lower (39.2%). Although specific CTP and MELD scores for responders were not provided, the overall lower response rate could indicate a more challenging patient population or differences in treatment efficacy. In the study by Gupta et al. [14], the better response could be attributed to the higher dosage of albumin administered (1 gm/kg/day, with a maximum of 100 g/day) compared with our study, where patients were given 20 g of albumin per day. The purpose of using a lower dose of albumin was to evaluate the effect of a higher dose of terlipressin infusion initiated at admission instead of waiting for an albumin response at the end of 48 hours, where the deleterious effect of progressive renal impairment could cause an increase in morbidity and mortality. The difference in response rates between the infusion and bolus groups reported by Gupta et al. [14] and Cavalin et al. [12] indicates that the method of administration could influence treatment outcomes, and infusion seems to provide a better response rate. Early initiation of a terlipressin infusion at a dose of 4 mg/day along with a lower dose of albumin showed beneficial effects similar to those of a higher albumin dose (100 g/day).

In our study, the overall adverse event rate was 21.3%, with the most common events being abdominal pain (8%) and diarrhea (7.4%); of these, infusion had to be completely stopped in only one (0.73%) patient who developed arrhythmia. In a study by Gupta et al. [14], the infusion group had no adverse events, whereas the bolus group showed an adverse event rate of 31.2%, with diarrhea being the most common. This higher rate compared with that in our study suggests that the bolus method may be less tolerated. In the study by Cavallin et al. [12], the adverse event rate was 35.29%, with 20.59% of the patients experiencing severe events in the infusion group. The most common adverse event was angina pectoris, indicating significant cardiovascular risk. The adverse event rate was much higher (62.16%), with 42.24% experiencing severe events in the bolus group, the most common being circulatory overload. These findings suggest that the bolus method not only increases the overall adverse event rate but also the severity of events, highlighting the significant risks associated with this administration method. Wong et al. [13] reported a very high adverse event rate of 88%, but only 12% of them were severe. The most common adverse events were respiratory failure (10%) and abdominal pain (5%). This high rate of adverse events, albeit mostly non-severe, suggests the need for close monitoring and reconsideration of the treatment approach. Our study's adverse event rate was relatively low, particularly when compared with the much higher rates observed by Cavallin et al. [12] and Wong et al. [13]. The stark contrast in adverse event rates between infusion and bolus groups in the studies by Gupta et al. [14] and Cavallin et al. [12] underscore the importance of administration methods. Infusion seems to result in fewer and less severe adverse events than bolus administration.

Terlipressin infusion at a dose of 4 mg/day appears to have similar tolerability with a slight increase in adverse effects, with probable better outcomes compared with a lower infusion dosage of 2 mg/day, and may also decrease the cumulative dosage of terlipressin and albumin required in resource-limited settings.

Limitations

Our retrospective observational study lacked randomization and a comparator arm for bolus terlipressin administration. Albumin was administered at a fixed dosage of 20 g/day, as per the standard recommendation. The maximum dosage of terlipressin used in our study was 4 mg/day with a 1 mg bolus on day one, which is less than the maximal optimization dosage of 12 mg/day. There is limited data for comparison of terlipressin infusion at different doses.

## Conclusions

Terlipressin remains the most studied and widely used pharmacotherapy for the treatment of HRS-AKI. Our study provides a detailed analysis of the demographic, clinical, and safety profiles of patients with CLD undergoing treatment for HRS-AKI in a tertiary care hospital in South India. Terlipressin infusion has sustained effects on splanchnic hemodynamics with fewer severe adverse events. These findings emphasize the importance of optimizing treatment protocols: early initiation of terlipressin infusion at a higher dose (4 mg/day) with optimized albumin usage could decrease morbidity and mortality associated with HRS-AKI and minimize the adverse effects associated with terlipressin.
